# A Preliminary Zebrafish Model of *ACTA2* Deficiency Reveals Increased Larval Phenotype Burden and Suggests Reduced Adult Mutant Survival

**DOI:** 10.3390/genes17070808

**Published:** 2026-07-15

**Authors:** Mohammad A. Zafar, Lisa C. Harling, Yupeng Li, Nafiye B. Celik, Sandip K. Mukherjee, John Rizzo, Andrew Prendergast, John A. Elefteriades

**Affiliations:** 1 Aortic Institute at Yale-New Haven, Yale University School of Medicine, New Haven, CT 06519, USA; mohammad.zafar@yale.edu (M.A.Z.); lisa.harling@yale.edu (L.C.H.); nafiyebusra.celik@yale.edu (N.B.C.); sandip.mukherjee@yale.edu (S.K.M.); 2 Department of Political Science & Economics, Rowan University, Glassboro, NJ 08028, USA; liy@rowan.edu; 3 Program in Public Health, Stony Brook University, Stony Brook, NY 11794, USA; rizzologic@gmail.com; 4 Yale Zebrafish Research Core, Yale University School of Medicine, New Haven, CT 06511, USA; andrew.e.prendergast@yale.edu

**Keywords:** zebrafish, *ACTA2*, *acta2*, CRISPR/Cas9, genome editing, aorta, thoracic aortic aneurysm and dissection

## Abstract

Background: *ACTA2* encodes smooth muscle alpha-actin and is one of the most common genetic causes of inherited non-syndromic thoracic aortic aneurysm and dissection. Although murine models have provided important mechanistic insight, complementary vertebrate systems may enable more rapid in vivo phenotyping and future therapeutic screening. Methods: Utilizing a CRISPR/Cas9-based approach, we developed zebrafish *acta2* mutant models and assessed phenotypes at larval and adult stages. At 3 days post-fertilization, larvae were screened by brightfield microscopy for edema, axis defects, hemorrhage, and a composite endpoint (any phenotype) under basal conditions and after exposure to 0.2 mM epinephrine. Phenotype occurrence was summarized as group-specific proportions across a 2 × 2 design defined by genotype (wild-type vs. *acta2*-deficient mutant) and treatment (DMSO control vs. epinephrine), with prespecified pairwise comparisons using Pearson’s chi-squared tests. Pooled fish-level logistic regression models were also fit for each endpoint. Adult follow-up was performed by genotyping all available *acta2* fish at the facility and tracking staggered tank-level cohorts longitudinally. Results: Initial gross morphologic assessment did not reveal overt external phenotypic differences between heterozygous or homozygous *acta2* mutant larvae and wild-type controls. In larval analyses, the mutant genotype was associated with a greater burden of adverse phenotypes than treatment exposure. For the composite endpoint, mutant larvae demonstrated higher proportions than normal larvae under both control (28.3% vs. 17.4%, *p* = 0.007) and epinephrine conditions (26.9% vs. 17.4%, *p* = 0.023), whereas epinephrine did not significantly alter composite phenotype frequency within either genotype. Similar genotype-associated trends were observed for axis defects and hemorrhage. In pooled logistic regression, *acta2* deficiency was associated with increased odds of axis defects (OR 2.64, 95% CI 1.48–4.89, *p* = 0.001), hemorrhage (OR 2.40, 95% CI 1.09–5.68, *p* = 0.036), and the composite endpoint (OR 1.88, 95% CI 1.19–3.00, *p* = 0.008), whereas epinephrine exposure did not demonstrate a consistent independent effect across endpoints. Adult staggered follow-up showed greater attrition in homozygous mutant cohorts than in wild-type or heterozygous cohorts, with aggregated losses of 24%, 12%, and 3%, respectively. Conclusions: This preliminary zebrafish *acta2* model demonstrated that *acta2* deficiency is associated with increased adverse larval phenotype burden and suggested reduced long-term persistence of homozygous mutant fish in adulthood. The strongest signal in the current study was genotype-associated phenotype burden rather than a robust epinephrine-dependent effect. These findings support the feasibility of zebrafish-based *ACTA2* phenotyping while highlighting the need for more specific vascular endpoints, refined longitudinal follow-up, and future variant-specific modeling.

## 1. Introduction

Aortic aneurysm is the 20th leading cause of death in the United States, per 2021 CDC data. This is certainly an underestimate, as aneurysm deaths often are erroneously diagnosed as “heart attacks”. The natural history of thoracic aortic aneurysm (TAA) is characterized by progressive aortic dilation, eventually culminating in the lethal complications of dissection and rupture [1,2]. Timely prophylactic surgery once the aneurysmal aorta reaches a critical size threshold can prevent these lethal complications and restore normal survival, underlining the importance of understanding the natural behavior of the diseased aorta [3,4].

To date, 70 genes, with varying degrees of strength of causal evidence, have been identified as being associated with thoracic aortic aneurysm and dissection (TAAD) [5]. These genetic aberrations alter the natural history of the thoracic aorta, imparting gene-specific dissection and rupture risks, and therefore necessitating gene-unique intervention criteria. Marfan syndrome (caused by pathogenic *FBN1* gene mutations), Ehlers-Danlos syndrome (namely the vascular subtype caused by pathogenic *COL3A1* variants), and Loeys-Dietz syndrome (caused by TGF-β signaling pathway mutations) comprise a group of genetic disorders that predispose to syndromic TAAD, wherein patients exhibit overt multi-organ-system pathology, in addition to the aortic pathology [6,7,8]. Among non-syndromic TAAD (nsTAAD) patients, approximately one-fifth carry a family history of the disease. Furthermore, *ACTA2* gene mutations are the most frequent genetic aberration underlying inherited nsTAAD, accounting for up to 20% of the cases [9,10,11,12,13]. Clinical diagnosis of TAA disease is particularly challenging in nsTAAD patients, given the lack of external clinical signs and the silent nature of aortic disease. In fact, the majority of these genetic TAAD diagnoses come to light after potentially lethal dissection or rupture events [14].

The *ACTA2* gene encodes smooth muscle cell α-actin [11,15]. Mutations in this gene disrupt the smooth muscle cell elastin–contractile unit, whose normal functioning is critical for maintaining the structural integrity of the aortic wall [15]. Pathogenic *ACTA2* mutations are especially critical, imparting a 76% lifetime risk of a lethal aortic event [16]. These mutations cause acute aortic dissection without significant aortic dilation, often at diameters below guideline-recommended thresholds for prophylactic aortic intervention. Unfortunately, the median age of onset is only 36 years [9,16,17]. Therefore, interrogating mechanisms of *ACTA2* disease in vivo in animal models and exploring and validating therapies that could potentially favorably alter this malignant disease course is paramount.

Murine models have been used extensively to study genetic mutations causing TAAD, including Marfan, Loeys-Dietz, and *Acta2* mouse models [18,19,20,21,22,23,24,25]. Recently, our team has constructed zebrafish larval mutant models for a panel of known deleterious TAAD gene variants (*FBN1*, *COL1A2*, and *COL5A1*) and variants of unknown significance (*MIB1* and *EMILIN1*). We identified a dramatic pattern of structural anomalies: cerebral (intracranial hemorrhage), cardiac (cardiomegaly and edema), aortic (periaortic hemorrhage and diameter distortion), and skeletal (scoliosis and other axis defects), constituting an emerging zebrafish “aortic phenotype” [26,27]. In this study, utilizing a CRISPR/Cas9 approach, we extended our zebrafish experiments. Specifically, we developed *acta2* zebrafish models to screen for gross larval *acta2*-related structural anatomic disruptions. Furthermore, we explored the response of larval *acta2* zebrafish mutants to adrenergic stress induction, and we assessed adult *acta2* genotype-specific longitudinal follow-up.

## 2. Methods

### 2.1. General Zebrafish Maturation and Care Methodology

Adult AB zebrafish were housed in 3.0-L tanks under a 14-h light/10-h dark cycle with continuous water flow (Iwaki Aquatic, Holliston, MA, USA) and fed Gemma Micro 300 twice daily plus brine shrimp (*Artemia nauplii*) once daily (Skretting, Stavanger, Norway). Embryos were obtained by natural mating and maintained in E3 medium (5 mM NaCl [Millipore Sigma, St. Louis, MO, USA], 160 μM KCl [Millipore Sigma, St. Louis, MO, USA], 333 μM CaCl_2_ [Millipore Sigma, St. Louis, MO, USA], 416 μM MgCl_2_ [Avantor Performance Materials, Center Valley, PA, USA]) at 28.5 °C. When needed, fish were anesthetized with 0.16 mg/mL MS-222 (Syndel, Ferndale, WA, USA). Husbandry and care were modeled after Boston Children’s Hospital protocols [28,29]. All animal care and handling were carried out under the approval of the Yale Institutional Animal Care and Use Committee (IACUC) (Protocol#: 2019-20274).

### 2.2. CRISPR Reagents and Generation of Zebrafish Transient Mutants

**Figure 1:**
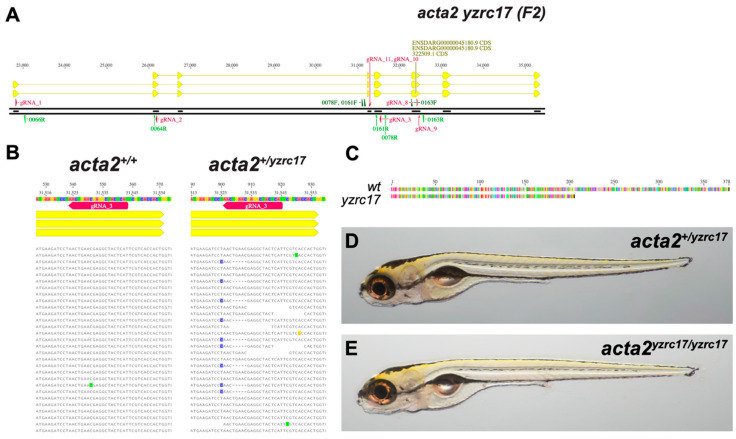
(**A**) Annotation of the utilized gRNAs in the zebrafish *acta2* gene. (**B**) Genetic code of both the *acta2*^+/+^ wild type (*wt*) and *acta2*^+*/yzrc17*^ heterozygote mutant, binding site of gRNA(3) visualized. (**C**) Visualization of the *wt* gene and the *yzrc17* mutant gene. (**D**,**E**) Normal morphology of the *acta2*^+/*yzrc17*^ heterozygote mutant and *acta2^yzrc17^*^/*yzrc17*^ homozygote mutant.

Four sequence-specific crRNAs targeting zebrafish *acta2* were designed using the CRISPRscan web tool as described by Moreno-Mateos et al. (Table 1, Figure 1) [30]. Each crRNA was annealed separately to trans-activating CRISPR (trac) RNA (Integrated DNA Technologies, Coralville, IA, USA) to form an RNA duplex stock (33 μM), which was then complexed with 10 μg/μL Alt-R S.p. Cas9 Nuclease V3 (IDT [Integrated DNA Technologies, Coralville, IA, USA]) in Cas9 buffer (20 mM HEPES, 150 mM KCl, pH 7.5) at a 1:1 molar ratio according to the manufacturer’s instructions. Separate crRNA-specific injection mixtures were prepared by supplementing each crRNA/Cas9 RNP complex with sterile 2% phenol red (Millipore Sigma, St. Louis, MO, USA) to a final phenol red concentration of 0.2%, yielding an approximate final RNP concentration of 3.3 μM. Zebrafish embryos were obtained at the 1–2 cell stage, and 1 nL of the described mixture was injected into each embryo using a Pneumatic PicoPump microinjection system (World Precision Instruments, Sarasota, FL, USA) under a dissecting microscope (Olympus, Needham, MA, USA). Embryos were allowed to recover in E3 medium. Teleosts, of which zebrafish are a member, underwent a whole-genome duplication event approximately 350 million years ago [31]. Consequently, they often have redundant paralogues of mammalian genes, which necessitate inactivating both copies. This method has previously been successfully applied for other diseases, including phenotypic screening of genes involved in the recovery from spinal cord injury or defective locomotor behaviors [32]. In our previous study, we observed an indel efficacy of 86%; for most gRNAs, we were unable to recover non-mutant sequences from individual clones taken from injected fish, suggesting a very high degree of mutation (reduced overall indel efficiency due to the apparent non-functionality of one gRNA) [26]. Embryos were injected with individual crRNAs targeting *acta2* (Table 1). The resulting F_0_ generation was evaluated for mutation efficiency and raised to adulthood. Mutation efficiency in larvae was assessed by isolating DNA from a small pool of F_0_ fish (10–20), followed by fragmentation analysis utilizing primer annealing to the RNP binding site. Adult F_0_ fish were outcrossed to generate F_1_ adults, which were sequenced and selected for indels generating frameshift mutations. Of these, two mutations generated by crRNA(3) were ultimately reserved and passaged to F_2_: *acta2*^yzrc15^ (insT) and *acta2*^yzrc17^ (del5). Sequencing in adult zebrafish was performed on DNA extracted from tail fin clippings. Both alleles appeared phenotypically similar to wild-type fish and survived to adulthood as homozygotes in F_2_ and beyond.

### 2.3. Zebrafish Maturation and Epinephrine Stress Induction

To impose adrenergic stress, larvae were exposed to epinephrine, beginning at 1 dpf. Fish were assigned to one of two conditions: E3 medium containing 0.2 mM epinephrine, 0.5% (*v*/*v*) dimethyl sulfoxide (DMSO [Millipore Sigma, St. Louis, MO, USA]), and 200 μM phenylthiourea (PTU [Millipore Sigma, St. Louis, MO, USA]), or control E3 medium containing 0.5% (*v*/*v*) DMSO and 200 μM PTU alone. DMSO was used to enhance membrane permeability, and PTU was included to inhibit pigmentation [33,34].

### 2.4. Brightfield Microscopy

At 3 dpf, injected larvae were screened by brightfield microscopy on an Olympus MVX-10 Macro Zoom microscope (Evident Scientific, Tokyo, Japan) for predefined gross phenotypes, including cerebral hemorrhage, aortic hemorrhage, scoliosis, axial curvature, cardiomegaly, and cardiac or ocular edema. Screening was performed blinded for *acta2*^−/−^ mutants vs. wild-type control.

### 2.5. Adult Genotype-Specific Follow-Up Across Staggered Cohorts

To further assess whether the *acta2* genotype was associated with reduced long-term viability, all available adult fish in the facility carrying the *acta2* genotype were genotyped, grouped by genotype, and followed longitudinally. Fish were maintained in multiple tanks, with each tank representing an independent cohort. Because fish were enrolled from existing facility populations rather than from a single synchronized birth cohort, cohorts entered follow-up at different ages, resulting in a staggered longitudinal design. Serial tank counts were recorded over time to assess genotype-specific loss of fish during follow-up.

For each tank, the number of fish present at the start and end of the observation interval was recorded, and proportional loss over the interval was calculated as the difference between starting and ending counts divided by the starting count. These analyses were intended as a descriptive longitudinal follow-up to compare relative attrition across wild-type, heterozygous, and homozygous mutant *acta2* fish, rather than as a formal time-to-event survival analysis.

## 3. Results

**Figure 2:**
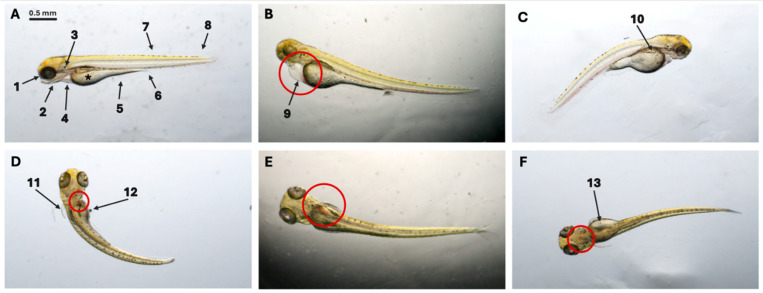
Photographs depicting zebrafish with (**A**) a normal phenotype, (**B**) cardiomegaly/cardiac edema (red circle), (**C**) an axis defect (sagittal plane; dorsal curvature), (**D**) scoliosis (coronal plane; lateral curvature) and small hemorrhage (red circle), (**E**) an aortic hemorrhage (red circle), and (**F**) cerebral hemorrhage (red circle). (*) yolk sac, (1) eye, (2) jaw, (3) otolith, (4) heart, (5) ventral fin, (6) cloaca, (7) dorsal fin, (8) caudal fin, (9) pericardium, (10) developing swim bladder, (11) pectoral fin, (12) air bubble, (13) melanocytes.

A total of 815 larval observations were analyzed across four groups (Table 2A,B): wild-type DMSO control (*n* = 220), wild-type plus epinephrine (*n* = 219), *acta2*^−/−^ DMSO control (*n* = 205), and *acta2*^−/−^ plus epinephrine (*n* = 171). Binary outcomes included edema (cardiomegaly), axis defects (scoliosis and axial curvature), hemorrhage (cerebral, aortic, and abnormalities in vascular morphology), and a composite adverse phenotype endpoint (any), defined as the presence of at least one of the three individual phenotypes (Figure 2).

Larval phenotyping was conducted on three separate experimental dates, designated replicates A–C. Differences in sample size across replicates primarily reflected variation in embryo production. Date-specific counts and proportions are provided in Table 2A.

**Figure 3:**
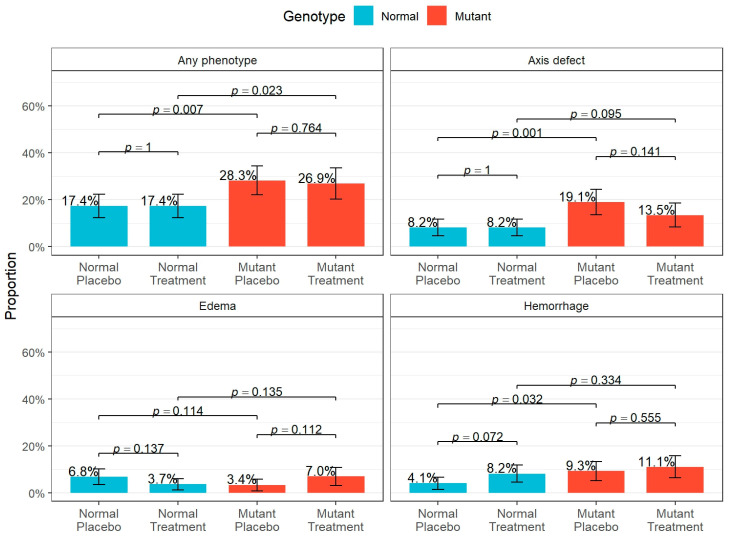
Group-specific proportions of adverse larval phenotypes by genotype and treatment. Phenotype occurrence is shown across the 2 × 2 study design defined by genotype (Normal vs. Mutant [*acta2*^−/−^]) and treatment (control [DMSO] vs. Epinephrine). Panels display the proportion of larvae with any phenotype, axis defects, edema, or hemorrhage. Bars represent group-specific proportions, and error bars indicate 95% confidence intervals. For each endpoint, proportions were calculated using the number of fish with observed data for that outcome as the denominator. Four prespecified pairwise comparisons were performed within each panel using Pearson’s chi-squared tests: Normal control vs. Normal Epinephrine, Mutant control vs. Mutant Epinephrine, Normal control vs. Mutant control, and Normal Epinephrine vs. Mutant Epinephrine. Exact *p*-values are shown above the corresponding brackets. Corresponding counts and proportions are provided in Table 2B.

Phenotype occurrence was summarized descriptively as group-specific proportions within the 2 × 2 design defined by genotype (Normal vs. Mutant) and treatment (Control vs. Epinephrine). For each phenotypic outcome (edema, axis defects, hemorrhage, and the composite endpoint (any)), the proportion was calculated using the number of fish with observed data for that specific outcome as the denominator. Group-specific proportions were visualized as bar plots with 95% confidence intervals (Table 2A,B and Figure 3).

For each phenotype, four prespecified pairwise comparisons were performed: Normal DMSO control vs. Normal Epinephrine, Mutant DMSO control vs. Mutant Epinephrine, Normal DMSO control vs. Mutant DMSO control, and Normal Epinephrine vs. Mutant Epinephrine. Differences in proportions were evaluated using Pearson’s chi-squared tests. Corresponding counts and proportions are provided in Table 2A,B.

The primary objective of the pooled analysis was to estimate the overall association of genotype and treatment exposure with adverse phenotype risk; therefore, data from all three experimental dates were combined for regression modeling. Pooled counts and proportions are provided in Table 2B. Separate binary logistic regression models were fit for each phenotype endpoint, with genotype (*acta2*^−/−^ vs. wild-type), treatment (epinephrine vs. DMSO control), and a genotype-by-treatment interaction term included as predictors. Wild-type larvae in the DMSO control condition served as the reference group. Regression coefficients, odds ratios (ORs), 95% confidence intervals (CIs), and *p* values were calculated for each model.

Initial gross morphologic assessment did not reveal overt external phenotypic differences between heterozygous or homozygous *acta2* mutant larvae and wild-type controls (Figure 1D,E).

Given this absence of an obvious baseline larval phenotype on brightfield examination, we next evaluated whether adrenergic stress exposure or longer-term adult follow-up would unmask *acta2*-associated abnormalities.

### 3.1. Gross Larval Phenotypes in acta2-Deficient Zebrafish Under Basal and Epinephrine-Stressed Conditions

Across the three experimental replicates, overall edema proportions were 6.8% in wild-type DMSO control larvae, 3.7% in normal epinephrine larvae, 3.4% in mutant (DMSO) control larvae, and 7.0% in mutant epinephrine larvae. Overall axis defect proportions were 8.2%, 8.2%, 19.1%, and 13.5%, respectively. Overall hemorrhage proportions were 4.1%, 8.2%, 9.3%, and 11.1%, respectively. For the composite ‘any’ phenotype endpoint, overall proportions were 17.4% in normal control larvae, 17.4% in normal epinephrine larvae, 28.3% in mutant control larvae, and 26.9% in mutant epinephrine larvae (Table 2A,B).

Proportion-based analyses further supported a genotype-associated increase in phenotype burden (Figure 3). For the composite endpoint (“any phenotype”), rates were identical in the two Normal groups (17.4% for both control and epinephrine; *p* = 1.000), whereas Mutant larvae showed significantly higher proportions than Normal larvae under both control (28.3% vs. 17.4%, *p* = 0.007) and epinephrine conditions (26.9% vs. 17.4%, *p* = 0.023). Within the Mutant group, epinephrine exposure did not significantly alter the composite phenotype rate (26.9% vs. 28.3%, *p* = 0.764).

A similar pattern was observed for axis defects. Axis defect frequency did not differ between Normal control and Normal epinephrine groups (8.2% vs. 8.2%, *p* = 1.000) but was significantly higher in Mutant larvae than in Normal larvae under control conditions (19.1% vs. 8.2%, *p* = 0.001). A nonsignificant trend toward a higher axis defect frequency was also observed in Mutant versus Normal larvae under epinephrine exposure (13.5% vs. 8.2%, *p* = 0.095). Epinephrine exposure did not significantly alter axis defect frequency within the Mutant group (13.5% vs. 19.1%, *p* = 0.141).

Edema remained infrequent across all four groups, ranging from 3.4% to 7.0%, and no pairwise comparison reached statistical significance.

For hemorrhage, a significant difference was observed between the Normal control and the Mutant control groups (4.1% vs. 9.3%, *p* = 0.032). In contrast, the corresponding comparison under epinephrine exposure was not significant (8.2% vs. 11.1%, *p* = 0.334). Treatment effects within genotype were likewise not significant for hemorrhage, although a trend toward higher hemorrhage frequency was observed in Normal larvae exposed to epinephrine compared with control (8.2% vs. 4.1%, *p* = 0.072).

In summary, these analyses indicate that the mutant *acta2*^−/−^ genotype was associated with a greater burden of axis defects, hemorrhage, and overall adverse phenotypes, whereas epinephrine exposure did not produce a consistent increase in adverse phenotype risk within either genotype under the present experimental conditions.

### 3.2. Logistic Regression Analysis of Adverse Larval Phenotypes

To further define the relationship between *acta2* genotype, epinephrine exposure, and adverse larval phenotypes, pooled fish-level logistic regression models were fit separately for edema, axis defect, hemorrhage, and the composite endpoint (any) (Table 3).

For edema, neither genotype alone nor treatment alone was significantly associated with edema frequency. The genotype-by-treatment interaction term was significant (OR 4.12, 95% CI 1.15–15.82, *p* = 0.033), indicating that the association between epinephrine exposure and edema might differ by genotype. However, given the wide confidence interval, the finding should be interpreted with caution.

For axis defects, *acta2*-deficient larvae had significantly greater odds of axis defects than wild-type larvae (OR 2.64, 95% CI 1.48–4.89, *p* = 0.001). Neither treatment (OR 1.00, 95% CI 0.50–1.99, *p* = 1.000) nor the genotype-by-treatment interaction (OR 0.66, 95% CI 0.27–1.59, *p* = 0.352) was significant, suggesting that axis defects were primarily associated with genotype rather than epinephrine exposure.

For hemorrhage, *acta2*-deficient larvae likewise had significantly higher odds of hemorrhage than wild-type larvae (OR 2.40, 95% CI 1.09–5.68, *p* = 0.036). Epinephrine exposure was associated with a trend toward increased hemorrhage that did not reach statistical significance (OR 2.10, 95% CI 0.94–5.00, *p* = 0.077), and the interaction term was not significant (OR 0.58, 95% CI 0.20–1.66, *p* = 0.319).

For the composite adverse phenotype endpoint, *acta2*-deficient larvae had significantly increased odds of demonstrating at least one adverse phenotype compared with wild-type larvae (OR 1.88, 95% CI 1.19–3.00, *p* = 0.008). In contrast, neither epinephrine treatment (OR 1.00, 95% CI 0.61–1.64, *p* = 1.000) nor the genotype-by-treatment interaction (OR 0.93, 95% CI 0.48–1.83, *p* = 0.839) was significant.

In summary, these regression analyses indicate that *acta2* deficiency was associated with increased odds of axis defects, hemorrhage, and the composite adverse phenotype endpoint, whereas epinephrine exposure did not demonstrate a consistent independent effect across phenotype endpoints. Evidence for a genotype-specific epinephrine treatment effect was limited, with a significant interaction observed only for edema.

### 3.3. Staggered Adult Follow-Up Suggests Increased Attrition in Homozygous acta2 Mutant Cohorts

As gross larval phenotyping may incompletely capture consequences of *acta2* disruption, particularly for vascular smooth muscle-dependent biology, we extended longitudinal follow-up into adulthood using stable mutant fish derived from heterozygous incrosses.

**Figure 4:**
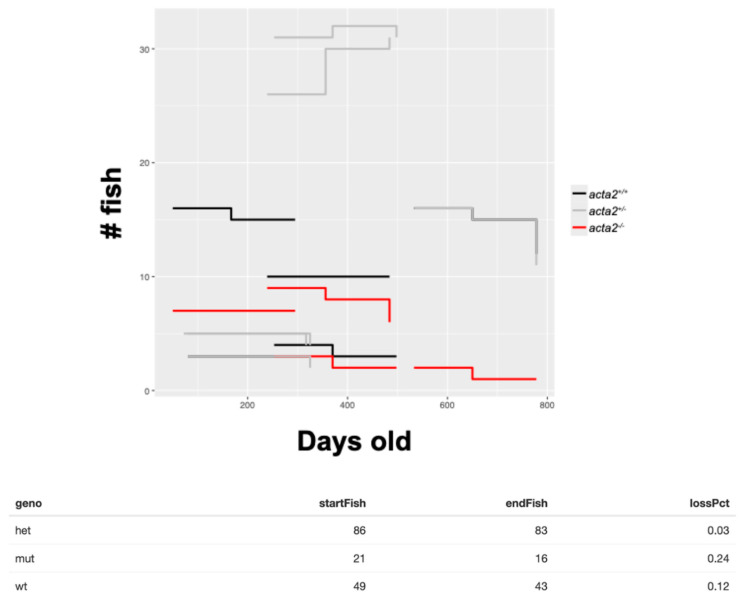
Longitudinal follow-up of staggered adult *acta2* cohorts by genotype. Each step line represents a separate tank cohort followed over time. The x-axis shows fish age in days, and the y-axis shows the number of fish present in each tank at each recorded count. Black lines indicate wild-type fish (*acta2*^+/+^; wt), gray lines indicate heterozygous fish (*acta2*^+/−^; het), and red lines indicate homozygous mutant fish (*acta2*^−/−^; mut). The table summarizes aggregate genotype-specific fish counts across all followed tanks. Starting fish count (startFish) denotes the total number of fish present at the first recorded observation for each genotype, Ending fish count (endFish) denotes the total number present at the final recorded observation, and “Loss” (lossPct) was calculated as starting count minus ending count, expressed as n (% of starting count). Homozygous mutant fish showed the greatest proportional loss during follow-up: 5 of 21 fish lost (24%), compared with 6 of 49 wild-type fish (12%) and 3 of 86 heterozygous fish (3%).

Across the observation period, homozygous mutant fish demonstrated the greatest proportional loss among the three genotype classes (Figure 4). Aggregated across tanks, heterozygous fish declined from 86 to 83 fish overall, corresponding to a 3% loss, whereas wild-type fish declined from 49 to 43 fish, corresponding to a 12% loss. In contrast, homozygous mutant fish declined from 21 to 16 fish, corresponding to a 24% loss over the follow-up interval. Thus, the relative reduction in fish number was greatest in mutant cohorts, approximately twofold higher than in wild-type cohorts and substantially greater than in heterozygous cohorts.

Visual inspection of the staggered cohort plot similarly suggested greater attrition among homozygous mutant tanks than among wild-type or heterozygous tanks. Several mutant cohorts showed progressive decline over time, including cohorts already at relatively advanced ages, whereas heterozygous cohorts were comparatively stable overall. One heterozygous tank showed an apparent increase in fish number during follow-up. Given that the subsequent two counts were closely aligned, this was considered most likely to reflect inaccuracy in the initial count rather than true gain of fish.

Overall, these descriptive longitudinal data suggest that homozygous *acta2* mutant fish may have reduced long-term viability relative to heterozygous and wild-type fish.

## 4. Discussion

In the present study, we developed and phenotyped a preliminary zebrafish *acta2* loss-of-function model across larval and adult stages. Several principal findings emerged. First, *acta2* mutant larvae did not exhibit overt baseline gross morphologic abnormalities on initial brightfield inspection. Second, larval *acta2* deficiency was associated with an increased burden of adverse phenotypes, namely axis defects, hemorrhage, and the composite endpoint of any adverse phenotype. Third, a logistic regression analysis revealed that genotype was the dominant driver of these adverse larval phenotype endpoints, whereas epinephrine exposure did not show a consistent independent effect across endpoints. Finally, long-term adult zebrafish follow-up revealed the greatest attrition in homozygous mutant *acta2* cohorts, suggesting a late-emerging viability phenotype that was not immediately apparent during early larval screening.

The lack of a clear baseline larval phenotype is not entirely unexpected when considered in the context of *ACTA2* biology. *ACTA2* encodes smooth muscle α-actin, the most abundant protein in smooth muscle cells and a core structural and functional component of the contractile apparatus in vascular smooth muscle cells [11]. Mouse studies have shown that *ACTA2* is not essential for cardiovascular development [22]. However, pathogenic mutations (most commonly heterozygous missense mutations) in *ACTA2* impair actin filament assembly, leading to reduced smooth muscle contraction and progressive vessel wall dysfunction [11]. In addition to thoracic aortic disease, *ACTA2* mutations are associated with early-onset coronary artery disease, stroke, and multisystem smooth muscle dysfunction syndromes [16]. Histologically, affected vessels show medial degeneration with smooth muscle cell loss, disorganization, and elastic fiber fragmentation; features that are hallmarks of human aortic disease. Clinically, *ACTA2* mutations are the most common cause of inherited nsTAAD, with patients presenting with acute dissections, often at relatively small aortic diameters and at younger ages than expected [16]. In that light, it is reasonable that a simple early-stage morphologic screen in zebrafish would not fully capture the downstream consequences of this biology, supporting the rationale for the subsequent stress-provocation and adult follow-up approaches.

In the larval stress experiments, *acta2*^−/−^ fish showed a higher burden of adverse phenotypes than wild-type controls, particularly for axis defects, hemorrhage, and the composite adverse event endpoint (Figure 3). These differences were observed in descriptive analyses and were further supported by the logistic regression analysis, wherein *acta2*^−/−^ mutant genotype was significantly associated with higher odds of axis defect, hemorrhage, and any adverse phenotype. The hemorrhage phenotype is biologically relevant because loss of *acta2* function would be expected to disrupt vascular structural integrity and potentially increase susceptibility to bleeding. The axis defect phenotype is less specific and may represent a broader developmental or structural abnormality in the zebrafish model, consistent with variable skeletal findings such as scoliosis reported in *ACTA2* families [35]. These findings suggest that the *acta2*^−/−^ mutation can cause overt abnormalities at the larval stage, even if they are not apparent on a simple baseline inspection. On the other hand, epinephrine treatment produced a much weaker signal than initially hypothesized. The composite phenotype endpoint was essentially unchanged by epinephrine treatment within both the wild-type *acta2* group (17.4% vs. 17.4%) and the *acta2*^−/−^ group (28.3% vs. 26.9%) (Figure 3), and the overall pattern was inconsistent. A significant mutant genotype by treatment interaction was observed for edema in the regression analysis (Table 3). However, this finding merits cautious interpretation given the low event counts and non-significant findings across other endpoints. Epinephrine exposure, therefore, did not unmask or amplify a larval *acta2*-associated phenotype under the present experimental conditions.

One possible explanation for these observations is that the epinephrine protocol employed in the current study did not impose sufficient or specific adrenergic stress to test the *acta2*^−/−^ induced vulnerability. Another is that the most relevant consequences of *acta2* disruption that recapitulate the human TAAD phenotype emerge later in development, when vascular smooth muscle organization and vessel wall mechanics are more mature. The adult zebrafish long-term follow-up data favor the latter possibility, wherein the homozygous mutant fish showed the greatest proportional loss during follow-up, declining from 21 to 16 fish overall (24% loss), compared with 49 to 43 fish in the wild-type group (12% loss) and 86 to 83 fish in the heterozygous group (3% loss). This aligns with what is known from human disease, where *ACTA2* mutations often manifest as vascular events in early or later adulthood rather than during development.

This study has several limitations. The larval phenotype endpoints used are broad morphologic features indicative of aortic pathology, as demonstrated in our prior work, but not specific to smooth muscle dysfunction. The pooled regression analysis, while useful for estimating overall effects, does not explicitly account for variability between experimental dates. Wide confidence intervals in our results warrant caution when interpreting results. In addition, the CRISPR/Cas9 approach used introduces complete gene disruption rather than recapitulating the heterozygous human *ACTA2* missense mutations.

## 5. Conclusions

This preliminary zebrafish *acta2* model demonstrates that *acta2* deficiency is associated with increased burden of adverse larval phenotypes. In contrast, epinephrine exposure did not show a consistent independent effect across adverse larval phenotypes. Adult follow-up further suggests reduced long-term persistence of homozygous *acta2*^−/−^ mutants, raising the possibility of a delayed viability phenotype that warrants further investigation. This work establishes stable *acta2* mutant zebrafish as a viable in vivo platform, while underlining the need to establish specific vascular endpoints, incorporate variant-specific knock-in models to recapitulate clinical *ACTA2*-associated vascular disease, and refine genotype-linked longitudinal adult zebrafish survival follow-up.

## Figures and Tables

**Table 1:** List of sequence-specific crRNAs used to target *acta2* in the zebrafish. Designed using the CRISPRscan web tool.

*acta2*	
crRNA (1)	GTGTAAGGCAGGCTTCGCCG
crRNA (2)	CGATGGGGTACTTGAGAGTC
crRNA (3)	ATGAGTAGCCTCGTTCAGTT
crRNA (4)	TGAGGTAGTCAGTGAGATCA

**Table 2:** (**A**,**B**): Replicate-specific and pooled counts and proportions of adverse larval phenotypes by genotype and treatment. Values are presented as the number of affected fish divided by the total number of fish evaluated in each replicate, with the corresponding percentage shown in parentheses. Groups are defined by genotype (wild-type [WT] vs. *acta2*-deficient mutant [KO]) and treatment (0.2 mM epinephrine vs. DMSO control). Replicates A, B, and C correspond to three independent experimental repeats. In Table 2B, Replicates A, B, and C were combined so that each genotype-treatment group was treated as a single pooled experiment. Differences in sample size across replicates and groups reflect variation in embryo production. The composite endpoint (Any phenotype) represents larvae exhibiting at least one adverse phenotype.

**(A)**					
Group	Replicate	Edema	Axis Defects	Hemorrhage	Any Phenotype
WT + epinephrine	A	0/24 (0.0%)	3/24 (12.5%)	1/24 (4.2%)	4/24 (16.7%)
WT + epinephrine	B	3/58 (5.2%)	5/58 (8.6%)	7/58 (12.1%)	12/58 (20.7%)
WT + epinephrine	C	5/137 (3.6%)	10/137 (7.3%)	10/137 (7.3%)	22/137 (16.1%)
WT + control	A	0/23 (0.0%)	2/23 (8.7%)	1/23 (4.3%)	2/23 (8.7%)
WT + control	B	3/58 (5.2%)	7/58 (12.1%)	3/58 (5.2%)	13/58 (22.4%)
WT + control	C	12/139 (8.6%)	9/138 (6.5%)	5/139 (3.6%)	23/138 (16.7%)
KO + epinephrine	A	1/29 (3.4%)	10/29 (34.5%)	1/29 (3.4%)	10/29 (34.5%)
KO + epinephrine	B	0/26 (0.0%)	0/26 (0.0%)	6/26 (23.1%)	6/26 (23.1%)
KO + epinephrine	C	11/116 (9.5%)	13/116 (11.2%)	12/116 (10.3%)	30/116 (25.9%)
KO + control	A	0/29 (0.0%)	3/29 (10.3%)	1/29 (3.4%)	4/29 (13.8%)
KO + control	B	0/50 (0.0%)	4/50 (8.0%)	2/50 (4.0%)	6/50 (12.0%)
KO + control	C	7/126 (5.6%)	32/126 (25.4%)	16/126 (12.7%)	48/126 (38.1%)
(**B**)					
Group	Replicate	Edema	Axis defects	Hemorrhage	Any phenotype
WT + epinephrine	Pooled (A–C)	8/219 (3.7%)	18/219 (8.2%)	18/219 (8.2%)	38/219 (17.4%)
WT + control	Pooled (A–C)	15/220 (6.8%)	18/219 (8.2%)	9/220 (4.1%)	38/219 (17.4%)
KO + epinephrine	Pooled (A–C)	12/171 (7.0%)	23/171 (13.5%)	19/171 (11.1%)	46/171 (26.9%)
KO + control	Pooled (A–C)	7/205 (3.4%)	39/205 (19.0%)	19/205 (9.3%)	58/205 (28.3%)

**Table 3:** Logistic regression analysis of adverse larval phenotypes in pooled zebrafish data. Separate binary logistic regression models were run for edema, axis defects, hemorrhage, and the composite endpoint (any). Predictors included genotype (*acta2*^−/−^ vs. wild-type), treatment (epinephrine vs. DMSO control), and a genotype-by-treatment interaction term. Wild-type larvae in the DMSO control condition served as the reference group. Odds ratios are presented with 95% confidence intervals.

Outcome	Term	Coefficient	Odds Ratio	95% CI	*p* Value
Edema	Intercept	−2.615	0.073	0.041–0.119	<0.001
	*acta2*^−/−^ Mutant	−0.727	0.483	0.181–1.171	0.120
	Epinephrine Treatment	−0.657	0.518	0.205–1.219	0.143
	Mutant × Treatment	1.416	4.120	1.154–15.817	0.033
Axis defect	Intercept	−2.413	0.090	0.053–0.141	<0.001
	*acta2*^−/−^ Mutant	0.971	2.639	1.476–4.885	0.001
	Epinephrine Treatment	0.000	1.000	0.503–1.988	1.000
	Mutant × Treatment	−0.419	0.657	0.270–1.589	0.352
Hemorrhage	Intercept	−3.155	0.043	0.020–0.078	<0.001
	*acta2*^−/−^ Mutant	0.873	2.395	1.085–5.678	0.036
	Epinephrine Treatment	0.742	2.100	0.944–5.000	0.077
	Mutant × Treatment	−0.540	0.583	0.196–1.662	0.319
Any	Intercept	−1.561	0.210	0.146–0.294	<0.001
	*acta2*^−/−^ Mutant	0.631	1.879	1.187–3.004	0.008
	Epinephrine Treatment	0.000	1.000	0.609–1.642	1.000
	Mutant × Treatment	−0.070	0.933	0.476–1.826	0.839

## Data Availability

The data presented in this study are available on request from the corresponding author.
